# Influence of the Presence of Choline Chloride on the Classical Mechanism of “Gelatinization” of Starch

**DOI:** 10.3390/polym13091509

**Published:** 2021-05-07

**Authors:** Doina Crucean, Bruno Pontoire, Gervaise Debucquet, Alain Le-Bail, Patricia Le-Bail

**Affiliations:** 1 ONIRIS, UMR 6144 GEPEA CNRS, F-44322 Nantes, France; doina.crucean@oniris-nantes.fr (D.C.); alain.lebail@oniris-nantes.fr (A.L.-B.); 2 INRAe, UR1268 Biopolymers Interactions Assemblies, BP 71627, F-44316 Nantes, France; bruno.pontoire@inrae.fr; 3 SFR IBSM 4202, BP 71627, F-44316 Nantes, France; 4 Audencia Business School, Human and Social Sciences, 8 Route de la Jonelière, BP 31222, F-44312 Nantes, France; gdebucquet@audencia.com

**Keywords:** allotropic transition, choline chloride, plasticizer, starch dissolution

## Abstract

The aim of this research is to contribute to a better understanding the destructuration of three native starches and a wheat flour in mixtures of water and choline chloride. Model systems have thus been defined to allow a better approach to hydrothermic transformations related to the interactions between choline chloride and starch. We have observed that choline chloride has an impact on the gelatinization of starch which corresponds to the stabilizing salts phenomenon. The depolymerization and dissolution of the starch have also been demonstrated and can there dominate the gelatinization. However, the results obtained in X-ray diffraction by heating cell have shown that the exotherm which appeared was not only related to the depolymerization of the starch, but that a stage of crystalline rearrangement of the starch coexisted with this phenomenon.

## 1. Introduction

Choline chloride, because of its unique nutrient functionality and its ability to enhance flavor, provides a new option as a partial substitute for sodium chloride in reformulations of processed foods to reduce their sodium content. To date, very few studies have explored the potential of choline chloride to act as a substitute for salt. Locke and Fielding were the first to identify the properties of choline chloride to improve the salty taste of food products [1,2,3].

Starch is a natural polymer with particular properties unlike those of traditional polymers. As a heterogeneous material, it has macromolecular structures bound in a granular superstructure.

When native starch granules are heated in water (excess of water conditions, around 60% water wb), their semicrystalline nature and three-dimensional architecture are gradually disrupted, resulting in a phase transition from the ordered granular structure to a disordered state in water, which is known as “gelatinization” [4,5]. Gelatinization is an irreversible process that includes several steps, such as granule swelling, native crystalline melting (loss of birefringence), and leaching of fractions of polymers or of polymers in particular amylose chains resulting in a molecular solubilization [6]. The gelatinization process is essential in the processing of foods. For improving the properties of starch and adding new functionalities, it is common to carry out the hydrothermal transformation of starch in environments containing substances other than water. Various plasticizers and additives for starch processing have been used, including polyols (glycerol, glycol, sorbitol, etc.) and nitrogen-containing compounds (urea, ammonium derived, and amines) [7,8]. An alternative class of materials known as ionic liquids, now commonly defined as salts, that melt below 100 °C has recently attracted much interest for the processing of biopolymers such as starch.

Choline chloride solubilized in water gives an ionic liquid. Several studies have shown the effects of ionic liquids (solvents and plasticizers) based on the effects of choline chloride on starch [9,10,11,12,13,14]. A study conducted by Decaen et al. highlights the plasticization of starch by ionic liquids based on an examination of choline [15]. The destructuration of native maize starch in mixtures of water and ionic liquids containing choline cations was studied in dynamic heating conditions, combining calorimetry, rheology, microscopy, and chromatographic techniques by Sciarini et al. [16].

This work presents the mechanism of choline chloride penetration into the starch grain and its impact on gelatinization and the structural evolution of starch during heating kinetics.

## 2. Materials and Methods

### 2.1. Materials

Wheat flour (type 65) was supplied by Evelia, La Varenne, France. Wheat starch was purchased from Loryma GmbH (Zwingenberg, Germany), and waxy corn starch was purchased from Roquette Italia SpA (Cassano Spinola, Italy). Potato starch and choline chloride (C_5_H_14_ClNO) were supplied by Sigma-Aldrich (France).

### 2.2. Sample Preparations

The measurements were carried out on wheat flour (F_w_) and wheat, potato, and waxy corn starch (S_w_, S_p_, and S_wc_, respectively) solubilized in an aqueous solution containing different concentrations of choline chloride.

The model systems studied being composed of three ingredients (flour, chlorine choline, and water) and were presented under the form of Equation (1) with F_x_, Cc_y_, and W_z_ being the flour (or starch), choline chloride, and water content, respectively.

F_x_Cc_y_W_z_ (1)

A mixing design has been used (1). The factors are the concentrations of each component of the mixture. Responses are expressed as a function of these concentrations with the sum of the mass fraction of each component being equal to 100 (x + y + z = 100). For each analysis, 5 g of mixture was prepared.

The mode of preparation (quoted ILS = “Ionic Liquid + Starch” for the rest of the paper) consisted in adding the choline chloride under the form of ionic liquid [15] (previous dissolution of Cc and water for one hour before starting the experiments); in that case, the script that was used was “(Cc + W)”, and the ionic liquid was then added to the flour. These systems were written with the following script: F_x_[Cc_y_W_z_].

All analyses were repeated in triplicate. A statistical analysis was performed with variance analysis (*p* < 0.005) to detect significant differences.

### 2.3. Methods

#### 2.3.1. Differential Scanning Calorimetry Measurements

Samples weighing 500 mg were placed in stainless steel pans, and a reference cell was prepared by adding an amount of water equal to the volume of water in the sample cells. The cells were then sealed with a heat-resistant seal. The cell containing the sample was agitated (50 rpm at room temperature) for one hour and then placed in the oven of the appliance. The pans were heated at a rate of 1 °C/min from 10 to 120 °C by using the SETARAM microcalorimeter (µDSC) III (France). Scans were run on the following starch suspensions: S_P20_[Cc_x_W_y_], S_W20_[Cc_x_W_y_], S_WC20_[Cc_x_W_y_], and F_W20_[Cc_x_W_y_]. All measurements were made at least in triplicate.

Thermal transitions were defined as T_Ge_ (peak temperature gelatinization), and ΔH_Ge_ denotes the transition enthalpies for different endotherms. The enthalpies were determined by endotherm integration, and all traces were normalized to 1 mg of dried sample. The partial enthalpies were calculated from the onset of the endotherm to the end (every 1 °C).

#### 2.3.2. X-ray Diffraction

The samples containing flour and starch suspended in [Cc_56_W_24_] were prepared as follows: S_WC20_[Cc_56_W_24_], S_P20_[Cc_56_W_24_], S_W20_[Cc_56_W_24_], and F_W20_[Cc_56_W_24_]. The appropriate amounts of choline chloride, water (when required), and starch were weighed. The same formulations without choline chloride were studied: S_WC45_W_55_, S_P45_W_55_, S_W45_W_55_, and F_W45_W_55_, corresponding to the same F/W ratio as the previous mixtures.

The samples were examined by wide-angle (WAX) X-ray diffraction. The measurements were performed using a D8 Discover spectrometer from Bruker-AXS (Karlsruhe, Germany). Cu Kα_1_ radiation, produced in a sealed tube at 40 kV and 40 mA, was selected and parallelized using a double Gobël mirror parallel optics system and collimated to produce a 500 µm beam diameter with sample alignment by a microscopic video and laser. The data were monitored by a VANTEC 500 2D detector (Bruker, Karlsruhe, Germany) for 10 min and normalized.

The sample was placed in a capillary with a diameter of 1.5 mm, and a second capillary was introduced into the first to avoid loss of water during the rise in temperature; the two capillaries were sealed. The as-prepared capillary was placed in a heating stage HFS91 (Linkam, Tadworth, UK). The detector was positioned at a focusing distance of 8.6 cm from the sample surface. It was in direct beam position [17]. The heating kinetic applied to the sample was 1 °C/min from 20 to 120 °C. Every 10 °C, a plateau of 10 min was introduced to enable the acquisition of the diffraction spectrum.

## 3. Results and Discussion

### 3.1. Gelatinization of Different Starches

The thermograms of wheat flour (F_W_), wheat starch (S_W_), potato starch (S_P_), and waxy-corn starch (S_WC_) in excess water (W = 80%) should serve as a reference and allow comparison with the F_20_[Cc_x_W_y_] systems, i.e., when choline chloride is added to the aqueous phase. All the samples exhibited an endotherm on their thermograms, which corresponded to the gelatinization of the starch at the following temperatures: F_W_ 61.7 ± 0.2 °C, S_W_ 58.1 ± 0.1 °C, S_WC_ 70.8 ± 0.1 °C, and S_P_ 65.7 ± 0.1 °C (Table 1). The gelatinization temperatures of the different samples studied increased in the following order: T_Ge_ (S_W_) < T_Ge_ (F_W_) < T_Ge_ (S_P_) < T_Ge_ (S_WC_), whereas the gelatinization enthalpies increased in the following order: ΔH_Ge_ (F_W_) < ΔH_Ge_ (S_W_) < ΔH_Ge_ (S_WC_) < ΔH_Ge_ (S_P_).

The thermograms of the F_W_ and S_W_ samples showed a second endotherm characterized by the fusion of the amylose-endogenous lipid complexes formed during gelatinization [18,19]. The melting temperatures of the amylose-lipid complexes for S_W_ and F_W_ were 97 ± 1 °C and 92.3 ± 0.2 °C, respectively.

### 3.2. Influence of Choline Chloride on Starch Destructuration

Since choline chloride behaves as an ionic liquid when it is in the presence of water, its behavior during the heating of a starch granule suspension may strongly influence gelatinization. A study comparing the previously discussed aqueous-phase suspensions containing solubilized choline chloride was carried out.

Micro-DSC was used to study the physicochemical characteristics of the following suspensions: S_WC20_[Cc_x_W_y_]_,_ S_P20_[Cc_x_W_y_], S_W20_[Cc_x_W_y_], and F_W20_[Cc_x_W_y_].

**Figure 1:**
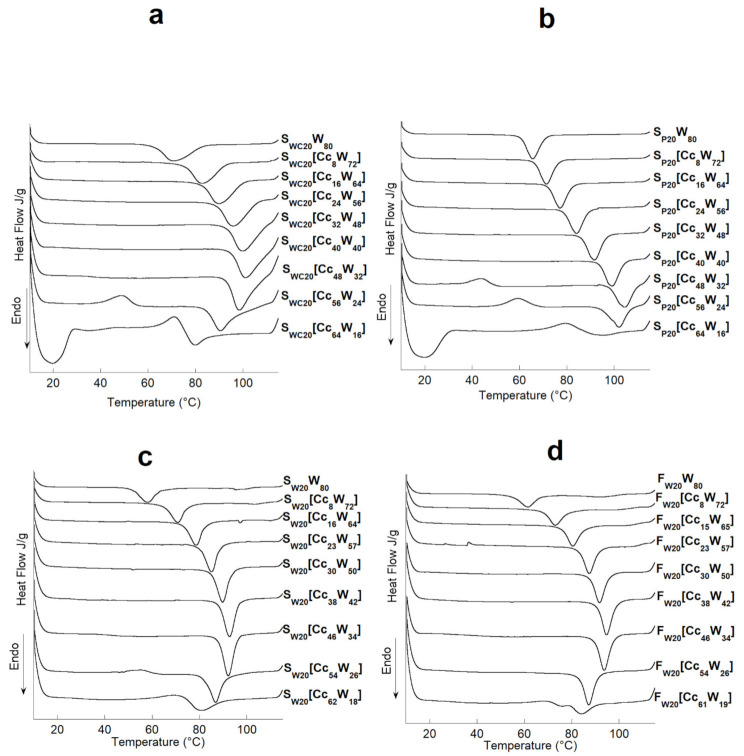
µDSC heating curves, 10–120 °C at 1 °C/min, of: (**a**) waxy-corn starch, (**b**) potato starch, (**c**) wheat starch, and (**d**) flour at different water and chlorine chloride contents.

The thermograms of the suspensions are shown in Figure 1, and their enthalpies ΔH_Ge_ and gelatinization temperatures T_Ge_ are shown in Table 1.

At low concentrations of Cc, the starch undergoes a typical gelatinization, represented by an endothermic transition. At the same concentration, the endothermic transition attributed to the fusion of endogenous amylose-lipid complexes is also observed for F_W_ and S_W_. When the concentration of Cc increases from 0 to 40% for S_WC_, S_W_, and F_W_ and from 0 to 48% for S_P_, the peak of gelatinization moves to a higher temperature and then decreases for all three starches and flour. The observed gelatinization temperature is always higher than that observed in pure water. However, beyond 40% for S_WC_, S_P_, and F_W_ and beyond 48% for S_W_, the gelatinization of the starch decreases with an increase in the B4 concentration. The same trend is observed for the enthalpies of gelatinization.

The increase in gelatinization temperature caused by Cc and the subsequent decrease with an increasing Cc level agree with the results of Chiotelli et al. for wheat and potato starches, Chungcharoen and Lund for rice starch, Jane for corn starch, and those of Ahmad and William and Ghani et al. for sargo starch [20,21,22,23,24].

The shift of the gelatinization temperature toward higher temperatures as well as the increase in the total enthalpy of gelatinization in the presence of Cc may be due to the reduction of the water activity in the starch/plasticizer solution, which causes an increase in the energy required for chemical and physical reactions involving water [25] Jane [22] claimed that the effect of salts on starch gelatinization follows the “Hofmeister” series, the increase in the gelatinization temperature follows the charge density order of the ions (LiCl > NaCl > KCl > RbCl), that is, the order of the “structure making” effect. However, at concentrations greater than 4 M, these salts decrease the onset temperature of gelatinization. Choline reacts as an “ion structure maker”.

**Figure 2:**
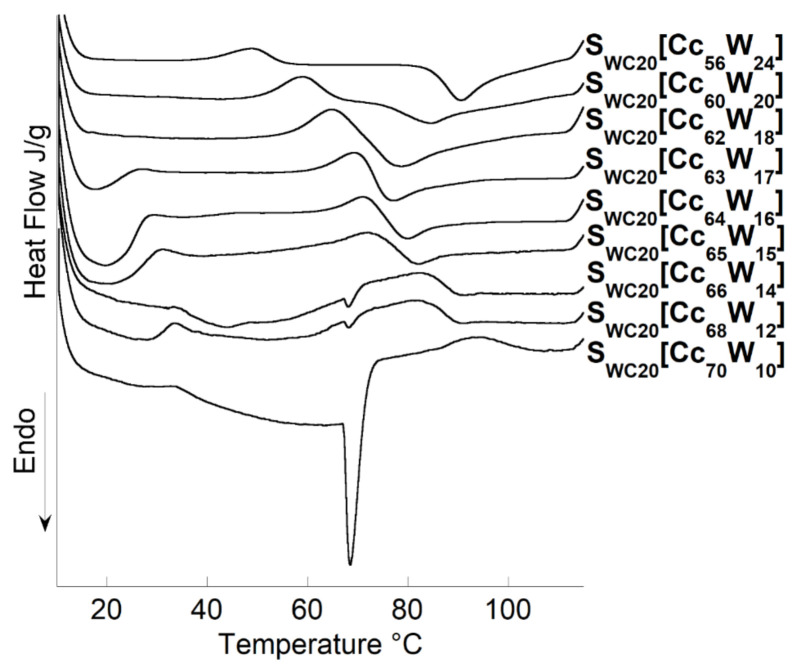
Microcalorimeter heating curves, 10–120 °C at 1 °C/min, of waxy-corn starch at different water, and choline chloride contents.

For high Cc concentrations, exothermic transitions were observed. The exotherms started at higher temperatures as the water content decreased, and the corresponding heat released (∆H) increased as well. There was a critical concentration, which depends on the type of starch used, for which both exothermic and endothermic transitions took place. For this concentration, both phenomena seem to happen at very close temperatures, at which they overlapped, resulting in a neutralization with no visible thermal effect. The same behavior was found by Sciarini et al. and Mateyawa et al. working with choline acetate and EMIMAc and by Koganti et al. using N-methyl morpholine N-oxide (NMMO) [12,16,26]. These authors attributed the exothermic transition to starch dissolution in these solvents. For low water contents (16%), the thermograms of S_P_ and S_WC_ show wide endotherms at low temperatures (19.7 °C). This phenomenon was not visible for S_W_ and F_W_. A DSC study was then carried out on the S_WC20_[Cc_x_W_y_] systems (with x included between 56 and 70 and y between 24 and 10) to understand this phenomenon (Figure 2).

The thermogram of pure Cc (data not shown) shows an endotherm near 73.6 ± 0.1 °C. With the addition of water, this endotherm moves to lower temperatures and undergoes a strong shift from the baseline until disappearing. In the presence of waxy maize starch (20%), the concentration of Cc was increased in steps of 1% (from 56 to 70%), and water was decreased likewise (from 24 to 10%), which produced an endotherm at low temperatures for the sample with 17% water.

During these investigations, the Cc was solubilized in water to form the plasticizer, and then the starch was added to the plasticizer. When the amount of water was sufficient, complete solubilization of the Cc was achieved. When the water concentration was less than 17%, the solubilization of Cc was partial, and we observed a solubilization endotherm that broadened with a decrease in the water content. For the 14% water sample, there was enough unsolubilized choline chloride for the allotropic change in choline chloride at 68 °C to be observed.

It was assumed that the solution (water + Cc) became more viscous with increasing Cc concentrations, resulting in more difficulty for the Cc solution to penetrate inside the starch granule; in turn, a shift to higher gelatinization temperatures was observed.

The effect of chlorine choline (Cc) on the gelatinization of starch is very different from that of NaCl observed in the literature. Indeed, if the gelatinization temperature follows the same trend when adding Cc or NaCl, the enthalpy of gelatinization shows the opposite behavior. The decrease in the total enthalpy of starch gelatinization at high NaCl concentrations suggests a destabilization of the ordered regions of the starch in the presence of NaCl. As explained by Chiotelli et al., sodium chloride may be hindered by polymer–polymer interactions in favor of water–polymer interactions, resulting in a lower enthalpy for fusion of organized regions. The addition of Cc causes a reorganization of the internal structure of the starch grain [20].

### 3.3. Penetration of the Plasticizer into the Starch Grain

To understand the penetration kinetics of the ionic liquid (Cc + water) in the starch granule, the cumulative enthalpy curves were calculated and reported as a function of temperature.

**Figure 3:**
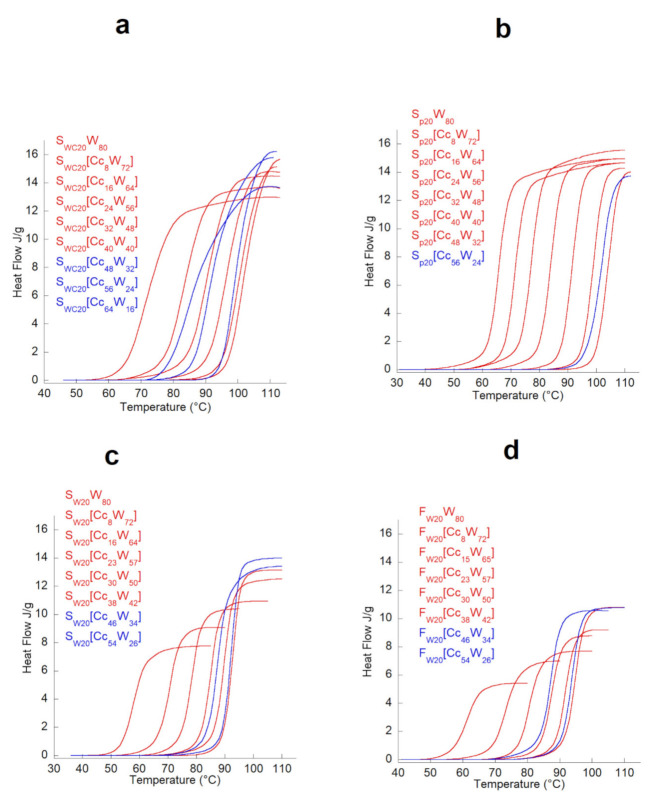
Curves of the partial enthalpy of gelatinization for all the plasticizer contents studied. (**a**) [S_WCx_Cc_y_]W_z_ systems; (**b**) [S_Px_Cc_y_]W_z_ system_s_; (**c**) [S_Wx_Cc_y_]W_z_ systems; (**d**) [F_Wx_Cc_y_]W_z_ systems.

The curves of the partial enthalpy of gelatinization for all the plasticizer contents studied are shown in Figure 3. This study presents the kinetics of the loss of ordered structure in starch for temperatures between 40 and 110 °C. An offset of the melting toward higher temperatures was observed when the Cc content increased; this was observed for the three starches and the flour.

The shapes of the curves shown in Figure 3 are rather different for S_P_ and S_W_: the melting of the potato starch ordered zones was more abrupt than it was for the wheat starch and the wheat flour. These observations are in good agreement with the results of Waigh et al. on the two-stage destructuration of S_W_ (crystallinity loss and helices destruction) [27]. Fannon, Hauber, and Bemiller showed that the S_P_ granules appear smooth on the surface by scanning electron microscopy, whereas cereal grains such as S_W_ have pores of about 100 nm at their surfaces, which are generated at the time of granule biosynthesis and cause a greater hydration sensitivity [28]. These observations may partly explain the differences in the plasticizer penetration into the granules for wheat starch and potato starch.

For potato starch, the total gelatinization enthalpy (ΔH) in the presence of Cc remained nearly constant at low concentrations of Cc but decreased slightly for higher concentrations. For wheat starch, the ΔH increased significantly as the concentration of Cc increased, to a greater extent than that for the potato starch. This considerable increase in the total enthalpy of gelatinization of wheat starch at high Cc concentrations suggests a better organization of the ordered regions of starch in the presence of Cc; on the other hand, when the concentration reached 40% for the Cc, the total enthalpy of the starch due to the fusion decreased perceptibly. The effect of high Cc concentrations on the gelatinization process was different for potato starch compared to waxy maize and wheat starch and wheat flour; it is assumed that this was due to the differences in the polymorphic structures.

The effect of Cc on the gelatinization of S_P_ is more pronounced in the first step of the gelatinization process (retardation of the loss of crystalline order), whereas for S_W_, F_W_, and S_WC_, Cc also affects the second stage (delay in the loss of molecular order) and the overall enthalpy of the transition. These results are again opposite to those found in the literature for potato starch and wheat starch in the presence of high salt concentrations (NaCl) [20].

### 3.4. Impact of the Formulation on Starch Gelatinization

As previously described, choline chloride in the absence of water is not considered as an ionic liquid. Therefore, it seemed interesting to observe the action of Cc on starch when Cc is no longer acting as a plasticizer, but when it enters into the formulation as dry matter, Cc is placed directly in competition with water in front of starch. The relevant suspensions were formulated as follows: the starch (or flour) was mixed with the choline chloride (dry powder), and the water was added to the mixture. The obtained mixes were quoted using the following script: [F_x_Cc_y_]W_z_. These mixtures were studied by μDSC.

The temperatures and enthalpies of starch gelatinization did not vary with the formulation. However, there was a marked increase in the solubilization/rearrangement exotherm when the choline chloride was used as an ionic liquid (data not shown). This observation is explained by the fact that the solubilization of Cc before deposition on the starch allowed better penetration into the grain.

**Figure 4:**
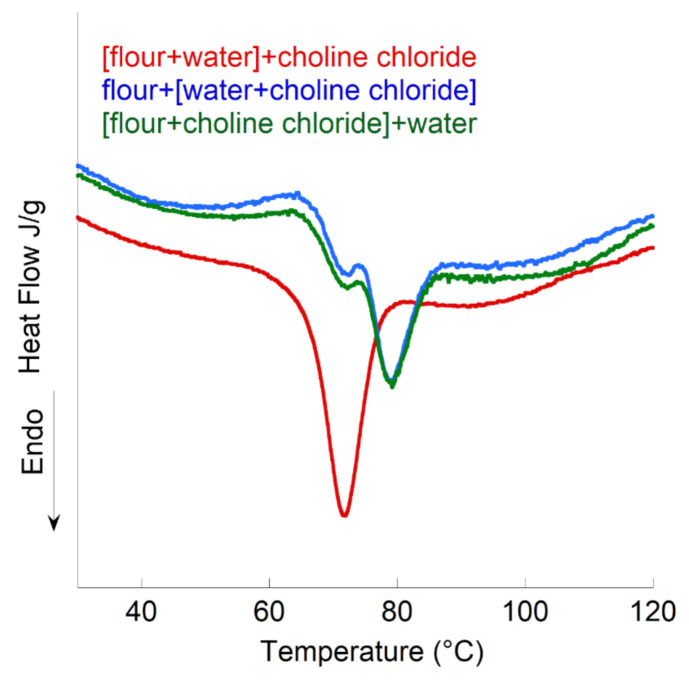
Comparative study of the thermograms of the systems [F_W20_Cc_64_]W_16_, F_W20_[Cc_64_W_16_], and [F_W20_W_16_]Cc_64_.

Another comparative study was carried out on the F_W20_Cc_62_W_18_ system, with three different formulations (Figure 4):

- Formulation 1: the wheat flour was mixed with water before the introduction of choline chloride for [F_W20_W_18_]Cc_62_.
- Formulation 2: choline chloride was mixed with water before being applied to the wheat flour for F_W20_[Cc_62_W_18_].
- Formulation 3: the wheat flour was mixed with choline chloride before the introduction of water for [F_W20_Cc_62_]W_18_.

The results clearly showed that when the water (in small quantities) is premixed with the flour, it becomes inaccessible to the choline chloride, causing gelatinization at 90 °C corresponding to a hydration of approximately 50%, which agrees with the starting hydration (following formula).

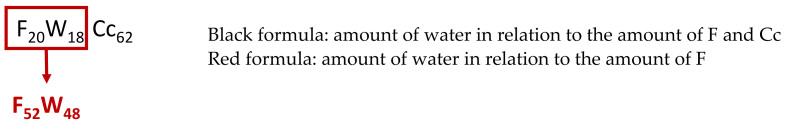



There is also a broad endotherm of allotropic change in Cc, suggesting that choline chloride has access to only a very small amount of water.

For the other two formulations, very similar results were obtained: namely, no change in the gelatinization temperature, a slightly more prominent exotherm for formulation 2, and a small endotherm associated with the allotropic change in Cc.

In this part, we have shown that the order of incorporation of water into the system can influence the gelatinization of the studied starches. Indeed, if the water is added to the starch first, it binds enough to the starch to become unavailable for Cc. On the other hand, if the water is first added to the choline chloride, the latter is solubilized before entering as an ionic liquid in the starch.

### 3.5. Evolution of the Structure of Different Starches in the Presence of Choline Chloride during Heating Kinetics

To understand the phenomena underlying the exothermic transitions observed with the microcalorimeter, measurements by an XRD heating cell were made.

**Figure 5:**
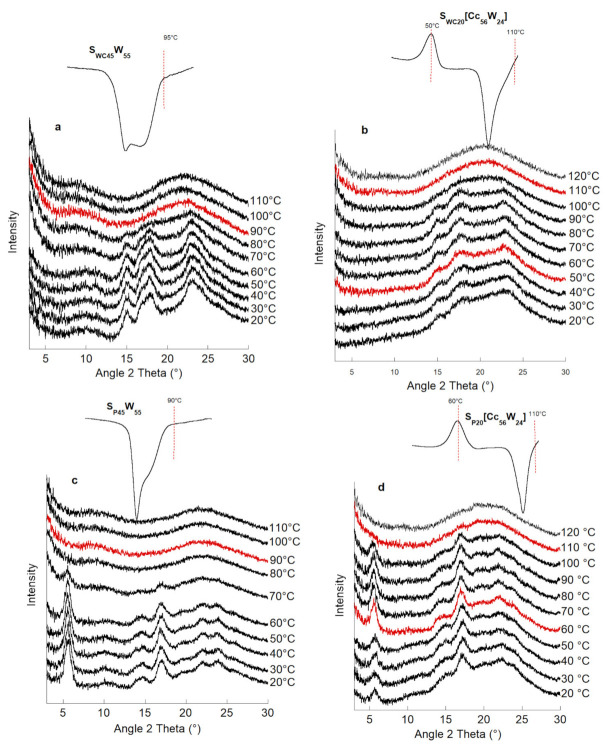
Evolution of the X-ray diffraction spectra during heating associated with the thermogram: waxy maize starch without Cc: S_WC45_W_55_ (**a**) and with Cc: S_WC20_[Cc_56_W_24_] (**b**); potato starch without Cc: S_P45_W_55_ (**c**) and with Cc: S_P20_[Cc_56_W_24_] (**d**).

Two types of systems were compared with and without Cc. The starch/flour and water concentrations were the same in both systems. The X-ray diffraction spectra of each of the suspensions of waxy corn starch and potato starch are collated in Figure 5.

S_WC_, S_W_, and F_W_ possess A-type polymorphic structures, and S_P_ possesses a B-type polymorphic structure. In the diffraction spectra of the S_W20_W_24_ (not shown), F_W20_W_24_ (not shown), and S_WC20_W_24_ systems, characteristic peaks of the A-type structure were identified. Spectra of the S_P20_W_24_ system showed characteristic peaks of the B-type structure.

A notable difference is seen in the evolution of the spectra during heating between the samples without Cc (Figure 5a,c) and those containing Cc (Figure 5b,d). Indeed, the peaks are more well defined in the systems without Cc, as with the addition of Cc at 20 °C. This confirms the previous conclusion: Cc causes a loss of crystalline order for all types of starch at room temperature. For the S_P45_W_55_ system, the type-B structure disappears at a temperature of 90 °C. However, for type-A systems, the structure collapses near 90 °C; this is consistent with Waigh’s observations [29]. When Cc was added to the systems, a collapse of structure A or B was observed at much higher temperatures, which agrees with the results obtained by DSC.

For the S_P20_[Cc_56_W_24_] system, an increase in the peak intensity occurred at 5.6° in 2Θ at a temperature of 60 °C, followed by the onset of collapse at 100 °C and the total disappearance of the structure at 110 °C. This phenomenon corresponds to the appearance of the exotherm on the thermogram from the DSC study.

For systems S_WC20_[Cc_56_W_24_] and S_W20_[Cc_56_W_24_], the transition to an increase in crystallinity before melting is much less visible, and it is even absent for the F_W20_[Cc_56_W_24_] system. This explains the absence of the exotherm for the F_W20_[Cc_56_W_24_] system. In the literature, the exotherm is related to the solubilization of the starch in the plasticizer. However, during the X-ray diffraction study, we showed that the exotherm also corresponded to the increase in the intensity of the diffraction peaks and therefore to the increase in the crystallinity of the starch before its solubilization and then its melting.

**Figure 6:**
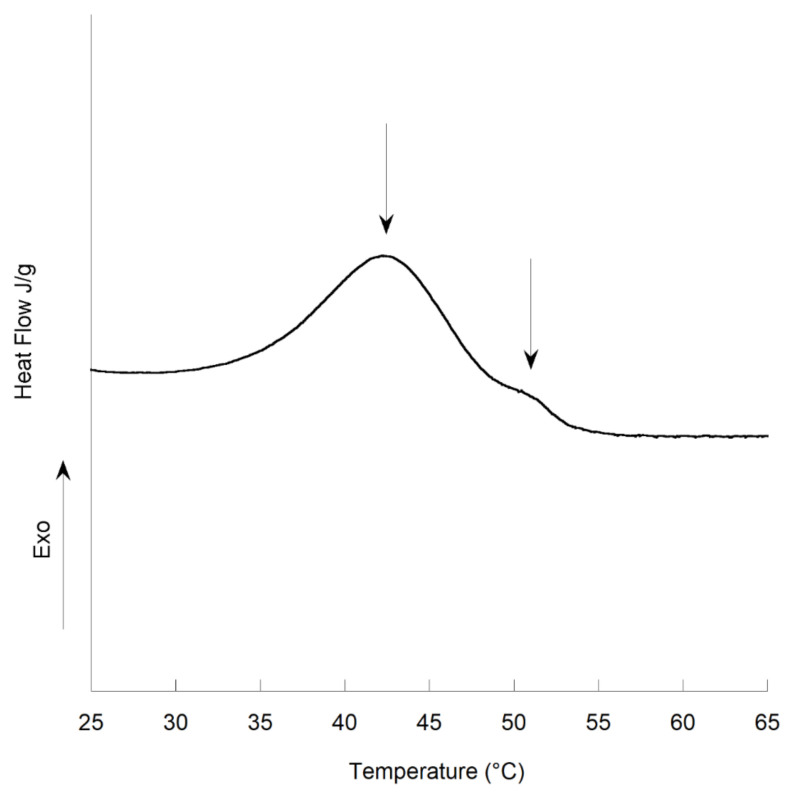
µDSC heating curves, 20–120 °C at 0.1 °C/min, for S_P20_[Cc_56_W_24_].

To complete this work, a μDSC study was conducted on the S_P20_[Cc_56_W_24_] system at low rates (0.1 °C/min). Two peaks were clearly observed (Figure 6), indicating that more than one phenomenon is responsible for the exothermic transition observed.

## 4. Conclusions

The starch/water system shows a gelatinization accompanied or unaccompanied by a fusion, according to the water content of the matrix. The choline chloride/water system possesses the characteristics of an ionic liquid and exhibits an allotropic change at low water concentrations and solubilization for water contents greater than 30%.

Choline chloride is an ionic compound, which, like NaCl, has a “structure making” effect: that is, it increases the viscosity of the aqueous solution while decreasing the water fraction with high mobility. As a result, the gelatinization temperatures are displaced to higher temperatures. In contrast to the addition of NaCl, the addition of choline chloride entails a significant increase in the gelatinization enthalpy, which suggests stabilization or better organization of the ordered regions of the starch in the presence of choline chloride.

An X-ray diffraction study in a heating cell demonstrated the crystalline rearrangement of the structure of the starch grain, which takes place simultaneously with the solubilization phenomenon of amylose.

## Figures and Tables

**Table 1:** Enthalpies (ΔHGe, J/g) and gelatinization temperatures (TGe, °C) of different flour/starch and plasticizer model systems. Enthalpies were calculated on flour or starch total dry basis.

x,y	Waxy-Corn Starch S_WC20_[Cc_x_W_y_]		Potato Starch S_P20_[Cc_x_W_y_]		Wheat Starch S_W20_[Cc_x_W_y_]		Wheat Flour F_W20_[Cc_x_W_y_]	
	ΔH_Ge_, J/g	T_Ge_, °C	ΔH_Ge_, J/g	T_Ge_, °C	ΔH_Ge_, J/g	T_Ge_, °C	ΔH_Ge_, J/g	T_Ge_, °C
**x = 0 y = 80**	10.8 ± 0.2	70.8 ± 0.1	12.0 ± 0.1	65.6 ± 0.1	7.2 ± 0.1	58.1 ± 0.1	5.2 ± 0.1	61.7 ± 0.2
**x = 8 y = 72**	12.4 ± 0.1	82.9 ± 0.1	12.2 ± 0.3	71.4 ± 0.1	8.4 ± 0.4	71.0 ± 0.1	6.3 ± 0.3	73.4 ± 0.4
**x = 16 y = 64**	13.3 ± 0.1	90.1 ± 0.2	12.8 ± 0.1	77.2 ± 0.1	8.8 ± 0.9	78.6 ± 0.1	6.7 ± 0.6	80.8 ± 0.3
**x = 24 y = 56**	14.9 ± 0.4	96.1 ± 0.2	13.4 ± 0.4	84.2 ± 0.1	10.3 ± 0.2	85.2 ± 0.1	7.7 ± 0.6	86.8 ± 0.7
**x = 32 y = 48**	14.9 ± 0.3	100.0 ± 0.1	13.6 ± 0.1	91.7 ± 0.1	11.4 ± 0.5	89.9 ± 0.1	8.6 ± 0.2	91.9 ± 0.2
**x = 40 y = 40**	14.9 ± 0.3	101.3 ± 0.1	14.2 ± 0.1	99.1 ± 0.1	12.2 ± 0.3	92.7 ± 0.1	9.2 ± 0.9	94.8 ± 0.1
**x = 48 y = 32**	16.8 ± 0.5	98.6 ± 0.1	14.0 ± 0.2	104.5 ± 0.1	13.0 ± 0.2	92.2 ± 0.1	9.7 ± 0.2	93.8 ± 0.1
**x = 56 y = 24**	16.1 ± 0.1	90.7 ± 0.1	13.5 ± 0.3	102.1 ± 0.1	12.0 ± 0.2	87.1 ± 0.3	9.2 ± 0.6	87.4 ± 0.2
**x = 64 y = 16**	N/A	79.9 ± 0.2	N/A	95.5 ± 0.2	N/A	81.0 ± 0.6	N/A	84.5 ± 0.5

## Data Availability

Nothing to report.
